# Supplementary material for: Neutral ceramidase regulates breast cancer progression by metabolic programming of TREM2-associated macrophages
Source: Nat Commun. 2024 Feb 1;15:966. doi: 10.1038/s41467-024-45084-7 (PMC10834982; doi:10.1038/s41467-024-45084-7)
Supplement: Supplementary file 1 — Supplementary Information [file 41467_2024_45084_MOESM1_ESM.pdf]

Neutral ceramidase regulates breast cancer progression by metabolic programming of TREM2-associated macrophages

Rui Sun[1,2,3 #], Chao Lei[1,2 #], Zhishan Xu[1,2], Xuemei Gu[2], Liu Huang[4], Liang Chen[1,2], Yi Tan[5], Min Peng[3], Kavitha Yaddanapudi[1, 2], Leah Siskind[6], Maiying Kong[2,7], Robert Mitchell[1,2], Jun Yan[1, 2] and Zhongbin Deng[1,2*]

1 Department of Surgery, Division of Immunotherapy, University of Louisville, KY, USA

2 Brown Cancer Center, University of Louisville, Louisville, KY40202, USA

3 Cancer Center, Renmin Hospital of Wuhan University, Wuhan, Hubei, 430060, P. R. China

4 Department of Oncology, Tongji Hospital, Tongji Medical College, Huazhong University of Science and Technology, Wuhan 430030, Hubei, P.R. China.

5 Department of Pediatrics, University of Louisville, Louisville, KY, USA

6 Department of Pharmacology & Toxicology, University of Louisville, Louisville, KY 40202, USA

7 Department of Bioinformatics and Biostatistics, University of Louisville, Louisville, KY, USA

#: These authors contributed equally

* Address correspondence to:

Dr. Zhongbin Deng
Department of Surgery
Brown Cancer Center
University of Louisville
CTRB 311
505 South Hancock Street
Louisville, KY 40202
E-mail address: z0deng01@louisville.edu

This Supplementary Information file includes: Supplementary Figures 1-8 and Supplementary table 1.

Supplementary Figure 1

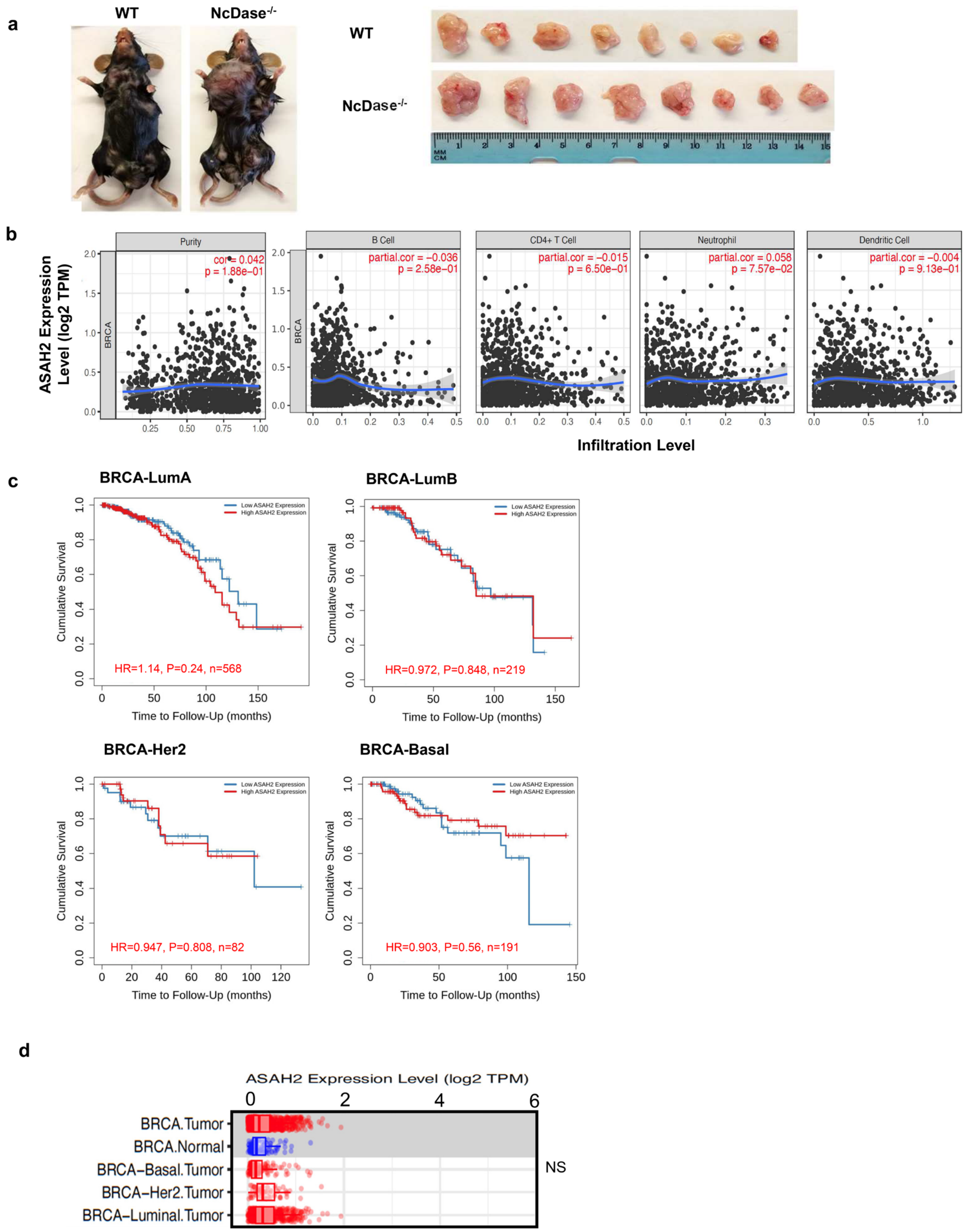

Supplementary Figure 1. Deletion of NcDase in the MMTV-PyMT model promotes tumor progression.

(**a**) Representative images of WT PyMT mice (n=21) or NcDase$^{-/-}$ PyMT mice (n=19) with tumors.
(**b**) Correlation of NcDase expression with tumor purity and infiltration level of indicated cell types in breast cancer samples. Data were from the TIMER 2.0 web platform (n=1,100). Spearman's correlation coefficients and P values are shown. TPM, transcript count per million reads.
(**c**) Kaplan-Meier curves representing overall survival for the subtypes of breast cancer patients related to high versus low NcDase expression as determined by CIBERSORT. P value was determined using the Logrank test and high and low expression levels were stratified by median value.
(**d**) NcDase (*ASAH2)* mRNA expression in tumor microarrays from patients with BRCA from the TCGA database. Wilcoxon test. NS: No significant difference.
(c-d) Tumor n=1,100 samples; normal n=112 samples; basal n=191 samples; Her2 n=82 samples; luminal A n=568 samples, and luminal B n = 219 samples.

Supplementary Figure 2

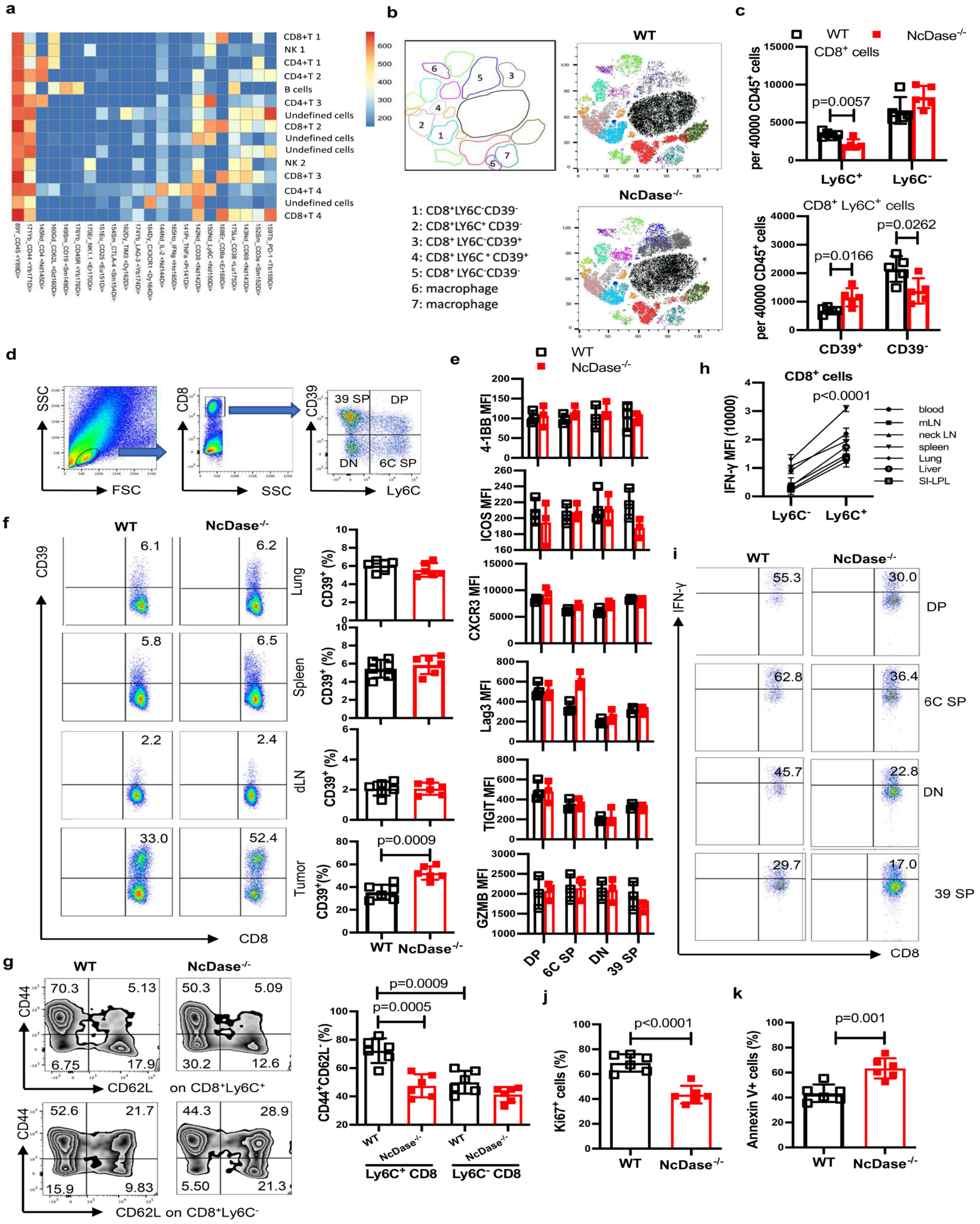

Supplementary Figure 2. Analysis of immune profiles within tumors based on CyTOF analysis.

(**a**) An in-depth T cell population analysis using FlowSOM clustering annotated into five final T cell subtypes is shown as a normalized expression heatmap. Data are representative of three independent experiments.

(**b-c**) t-SNE plot (b) and cell number (c) of clusters of the indicated CD8 T cell subsets. Forty thousand cells are displayed in each t-SNE plot. Images made with CyTOF data. n = 5 independent biological samples.

(**d**) Representative flow plots and gating strategies.

(**e**) CD8 T cells from MMTV-PyMT tumors were analyzed by flow cytometry for expression of the indicated populations: $CD39^+Ly6C^-$ (39 SP), $CD39^+Ly6C^+$ (DP), $CD39^-Ly6C^-$ (DN), and $CD39^-Ly6C^+$ (6C SP). The average MFI ± SD for the levels of various proteins in $CD8^+$ T cell subpopulations are shown in bar graphs. n = 3 independent biological samples. MFI, mean fluorescence intensity.

(**f**) Flow cytometry analysis of $CD8^+$ $CD39^+$ T cells in the indicated tissues from WT PyMT mice and $NcDase^{-/-}$ PyMT mice. n = 6 independent biological samples.

(**g**) Flow cytometry analysis of effector T cells in $Ly6C^-$ or $Ly6C^+$ $CD8^+$ T cells in the tumor from WT PyMT mice and $NcDase^{-/-}$ PyMT mice. n = 6 independent biological samples.

(**h**) Flow cytometry analysis of the expression of IFN-γ in $Ly6C^-$ or $Ly6C^+$ $CD8^+$ T cells. n = 3 independent biological samples.

(**i**) Flow cytometry analysis of the expression of IFN-γ in $CD39^+Ly6C^-$ (39 SP), $CD39^+Ly6C^+$ (DP), $CD39^-Ly6C^-$ (DN) and $CD39^-Ly6C^+$ (6C SP) in $CD8^+$ T cells in tumor-infiltrating lymphocytes (TILs) of WT PyMT mice or $NcDase^{-/-}$ PyMT mice. Representative of n = 3 independent biological samples.

(**j-k**) Frequencies of Ki-67^+ (j) or Annexin V^+ (k) in exhausted $Ly6C^+CD39^+$ $CD8^+$ T cells. n = 6 independent biological samples.

Statistical comparisons were performed using two-way ANOVA with Sidak's multiple comparisons test (c, e, g), two-tailed unpaired t-test (f, j, k), two-tailed paired t-test (h). Error bars indicate mean ± SD. Source data are provided as a Source Data file.

Supplementary Figure 3

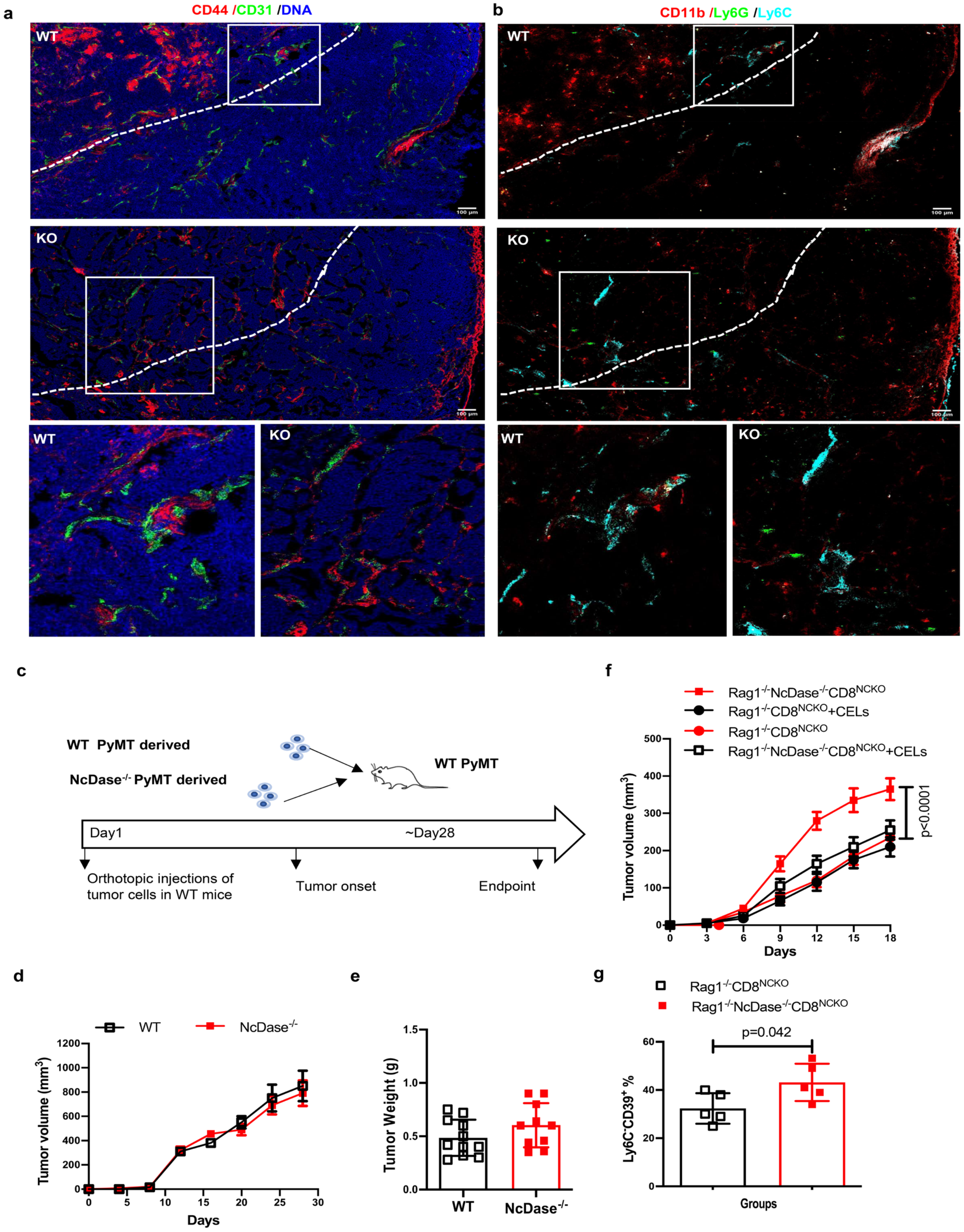

Supplementary Figure 3. NcDase regulates the function of tumor-associated myeloid cells.

(**a-b**) Representative IMC images of three independently stained tumor samples with the protein panel colored by different markers. A magnification of the indicated region (white box) from images of WT PyMT and NcDase$^{-/-}$ PyMT mice is shown on the bottom. Marker expression was false colored, and markers are indicated above each plot. A Gaussian blur (sigma = 0.65) was applied. Scale bars, 100 µm.
(**c**) Experimental setup of the in vivo experiment designed to assess the importance of NcDase in the tumor microenvironment, in which isolated WT PyMT tumor cells or NcDase$^{-/-}$ PyMT tumor cells were orthotopically injected into the mammary fat pad of WT PyMT mice without tumor at 7 weeks of age.
(**d-e**) Kaplan-Meier curves for tumor growth (d) and weight (e) after dissection of orthotopic tumors described in c. n=11 mice (WT) or 10 mice (NcDase$^{-/-}$).
(**f-g**) Rag1$^{-/-}$ mice or Rag1$^{-/-}$ NcDase$^{-/-}$ mice were reconstituted with 1×10^7 CD8$^+$ T cells from NcDase$^{-/-}$ mice (T cells were named CD8^{NCKO}) and treated with/without clodronate liposomes (CELs). Isolated WT PyMT tumor cells were orthotopically injected into the mammary fat pad. The kinetics of tumor cell growth were monitored (f) and the percentage of Ly6C$^-$CD39$^+$ in CD8$^+$ T cells in tumors was analyzed by flow cytometry (g). n = 5 independent biological samples.
Statistical comparisons were performed using two-tailed unpaired t-test (d, e, g), two-way ANOVA with Sidak's multiple comparisons test (f). Error bars indicate mean ± SD. Source data are provided as a Source Data file.

Supplementary Figure 4

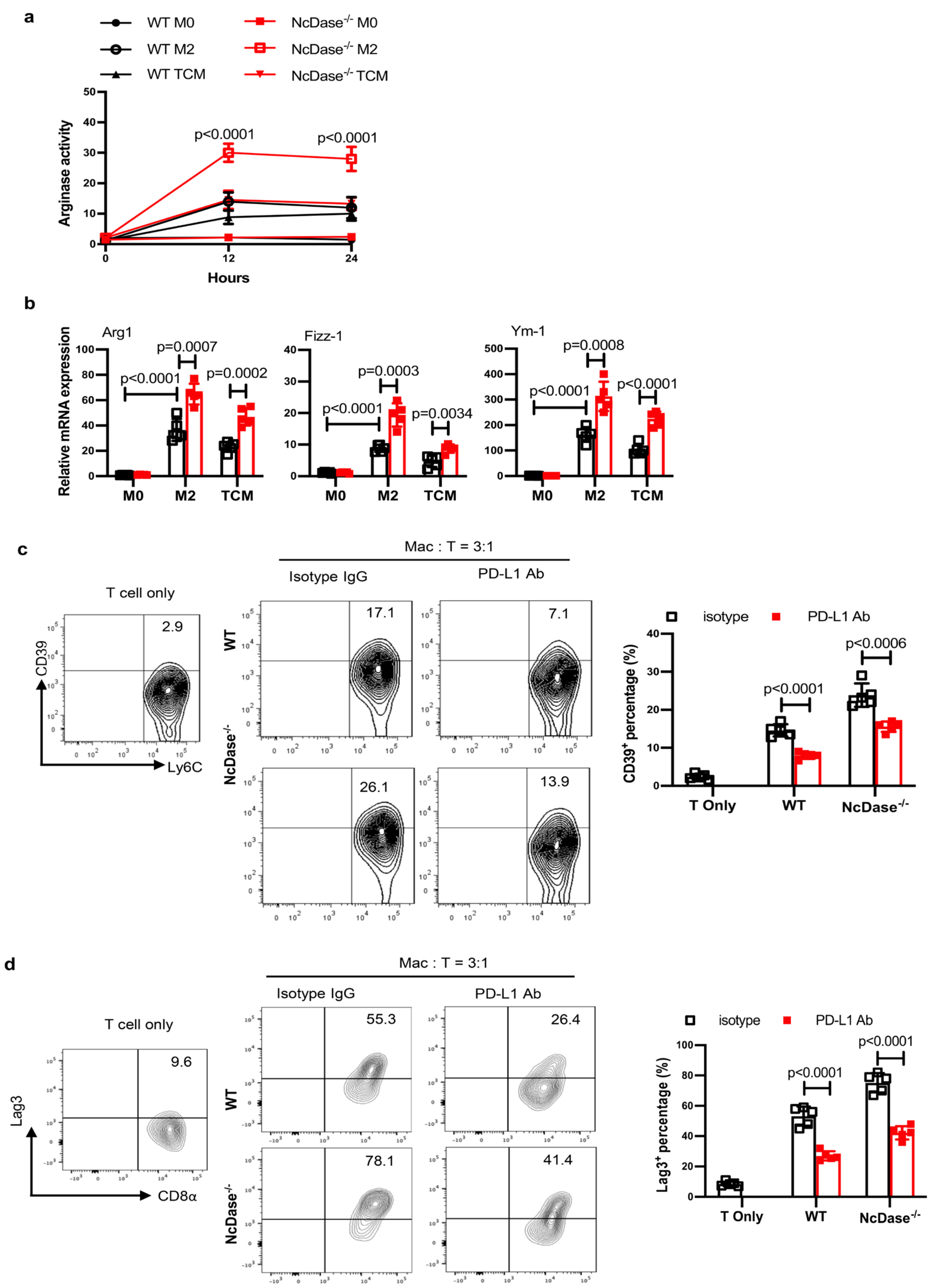

Supplementary Figure 4. NcDase deficient macrophages are associated with regulatory phenotype.

(**a**) Quantification of the activity of arginase on WT or NcDase$^{-/-}$ BMDMs differentiated from PBS, IL-4 or TCM. n = 5 independent biological experiments.

(**b**) Real-time PCR analysis of the mRNA levels of *Arg1*, *Fizz-1* and *Ym-1* genes on WT or NcDase$^{-/-}$ BMDMs differentiated from PBS, IL-4 or TCM. n = 5 independent biological samples.

(**c-d**) Quantification of the expression of CD39^{+} (c) or LAG3^{+} (d) on Ly6C^{+}CD8^{+} T cells that were co-cultured with TAMs sorted from WT PyMT or NcDase$^{-/-}$ PyMT tumors in the presence of PD-1 (50μg/ml) or IgG. n = 5 independent biological samples.

Statistical comparisons were performed using two-way ANOVA with Sidak's multiple comparisons test (a, b, c, d). Error bars indicate mean ± SD. Source data are provided as a Source Data file.

Supplementary Figure 5

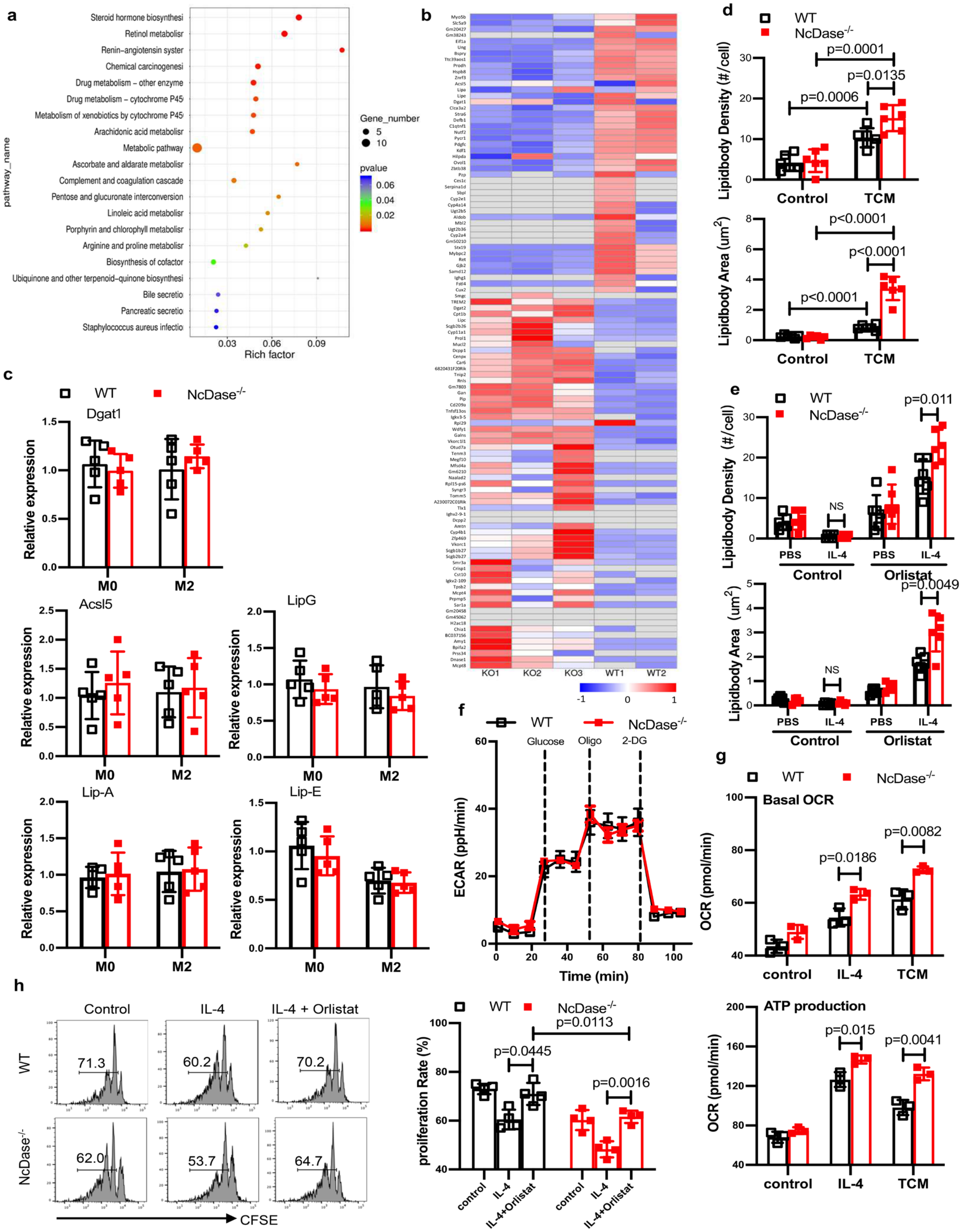

Supplementary Figure 5. Lipid accumulation and lipolysis for M2 activation is mediated by NcDase.

(a-b) Transcriptional profiles of TAMs sorted from WT PyMT (n = 3) or NcDase$^{-/-}$ PyMT mice (n = 2). GO enrichment analysis of genes defining the 20 most significant terms is represented in the accompanying bubble plot. Bubble colors represent the corrected p values. Bubble sizes indicate the numbers of genes (a). A heat map depicts expression levels of the major changes in genes including metabolic genes related to lipid droplets and lipolysis (b).

(**c**) Real-time PCR analyses of metabolic genes related to lipid droplets and lipolysis in M0 or M2 BMDMs derived from WT and NcDase$^{-/-}$ mice. n = 5 independent biological samples.

(**d**) Lipid droplets quantification using BODIPY493/503 staining was examined by confocal microscopy in PBS or TCM-derived BMDMs. The total number of LDs was counted per single cell. LD size defined by their cross-sectional area was calculated. n = 6 independent biological samples.

(**e**) Lipid quantification using BODIPY493/503 staining in PBS or IL-4-derived BMDMs with/without orlistat (100μM) treatment was examined by confocal microscopy. The total number of LDs was counted per single cell. LD size defined by their cross-sectional area was calculated. n = 6 independent biological samples. NS, not significant.

(**f**) ECAR of TAMs sorted from WT and NcDase$^{-/-}$ mice was measured using Seahorse technology and in response to the indicated drugs. n = 3 independent biological experiments.

(**g**) OCR of PBS, IL-4 or TCM-derived BMDMs from WT and NcDase$^{-/-}$ mice was measured using Seahorse technology. Histograms show basal OCR and ATP. n = 3 independent biological experiments.

(**h**) CFSE dilution and quantification representing the proliferation of CFSE$^{low/-}$CD8^{+} T cells after coculturing with BMDMs from WT and NcDase$^{-/-}$ mice. BMDMs were differentiated in either normal media, IL-4 or IL-4+orlistat. n = 4 independent biological experiments.

Statistical comparisons were performed using two-way ANOVA with Sidak's multiple comparisons test (c, d, e, g, h), Error bars indicate mean ± SD. NS represents no significant difference. Source data are provided as a Source Data file.

Supplementary Figure 6

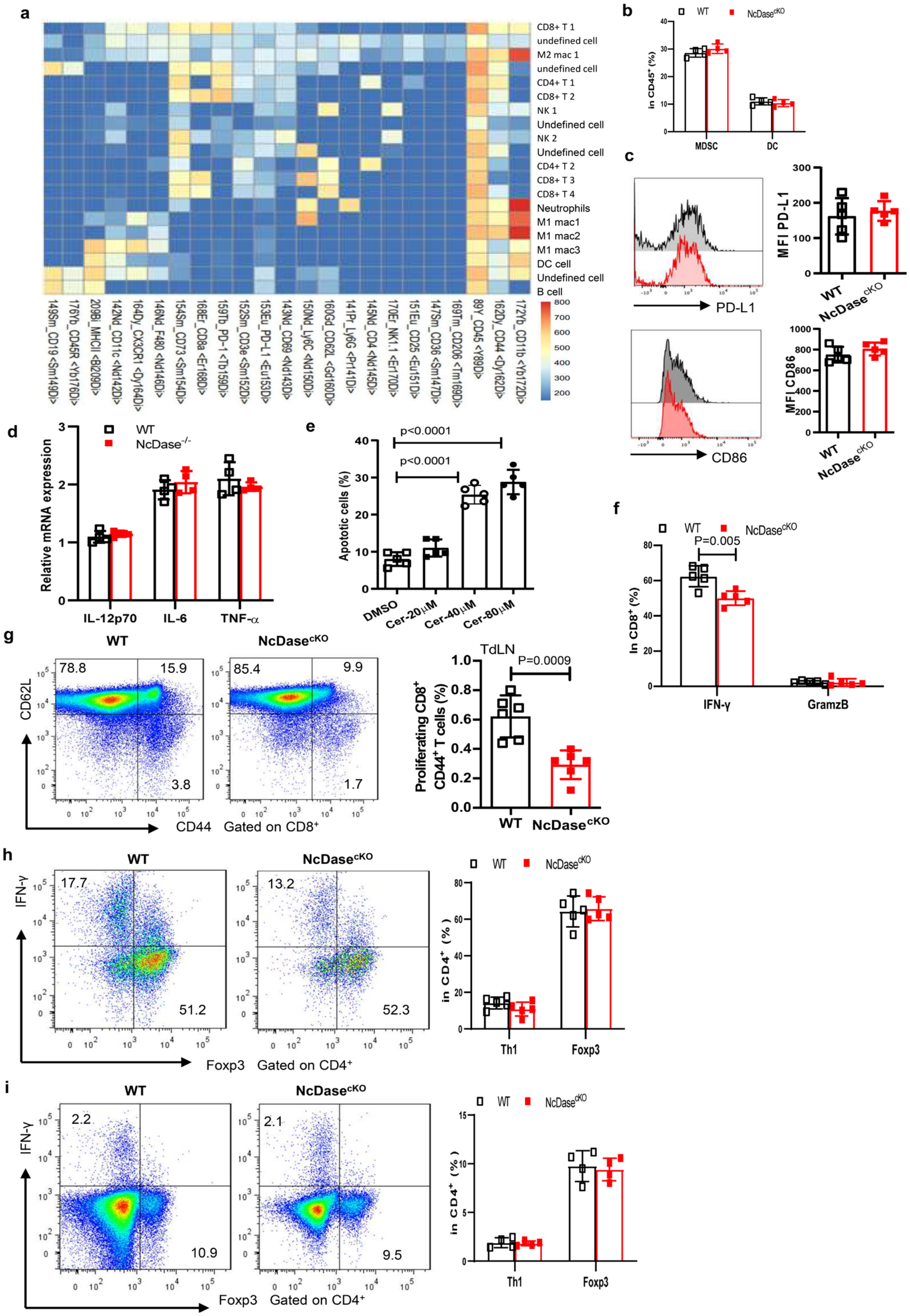

Supplementary Figure 6. Macrophage NcDase deficiency promotes the growth of breast cancer.

(**a**) CyTOF analysis based on FlowSOM clustering into 20 final major immune cell types for global immune profiling of the tumors is shown as a normalized expression heatmap. Data are representative of three independent experiments.

(**b**) Flow cytometry analysis of frequencies of $CD11b^+Gr\text{-}1^+$ MDSCs or $CD11c^+MHCII^+$ DCs in TILs isolated from E0771 tumors implanted. n=4 independent biological samples.

(**c**) Quantification of the expression of PD-L1 and CD86 on $CD11b^+CD11c^+$ DCs in the tumors. n=5 independent biological samples.

(**d**) Real-time PCR analysis of the mRNA levels of *IL-12*, *IL-6* and *TNF-α* genes on WT or $NcDase^{-/-}$ BMDC differentiated from TCM. n=4 independent biological samples.

(**e**) Exogenous ceramides induce DC apoptosis. Day 7 DC incubated with or without ceramide for 24 h. n=5 independent biological experiments.

(**f**) Frequencies of IFN-γ^+ $CD8^+$ and Granzyme B^+CD8^+ T cells in TILs isolated from E0771 tumors WT and myeloid NcDase knockout ($NcDase^{cKO}$) mice. n=5 independent biological samples.

(**g**) Representative dot plots and frequencies of proliferating $CD8^+$ effector T cells ($CD8^+$ $Ki67^+$ $CD44^{high}$ $CD62L^{low}$; pregated on live $CD45^+$ $CD3^+$ $CD8^+$ cells) in the TdLN of mice bearing EO771 tumors. n=6 independent biological samples.

(**h-i**) Representative dot plots and frequencies of $CD4^+$ IFN-γ^+ (Th1) and $CD4^+$ $Foxp3^+$ T cells in TILs (h) or TdLN (i) of mice bearing E0771 tumors. n=5 (h) or 4 (i) independent biological samples. Statistical comparisons were performed using two-tailed unpaired t-test (b-d, f-i), one-way ANOVA with Tukey's multiple comparisons test (e). Error bars indicate mean ± SD. Source data are provided as a Source Data file.

Supplementary Figure 7

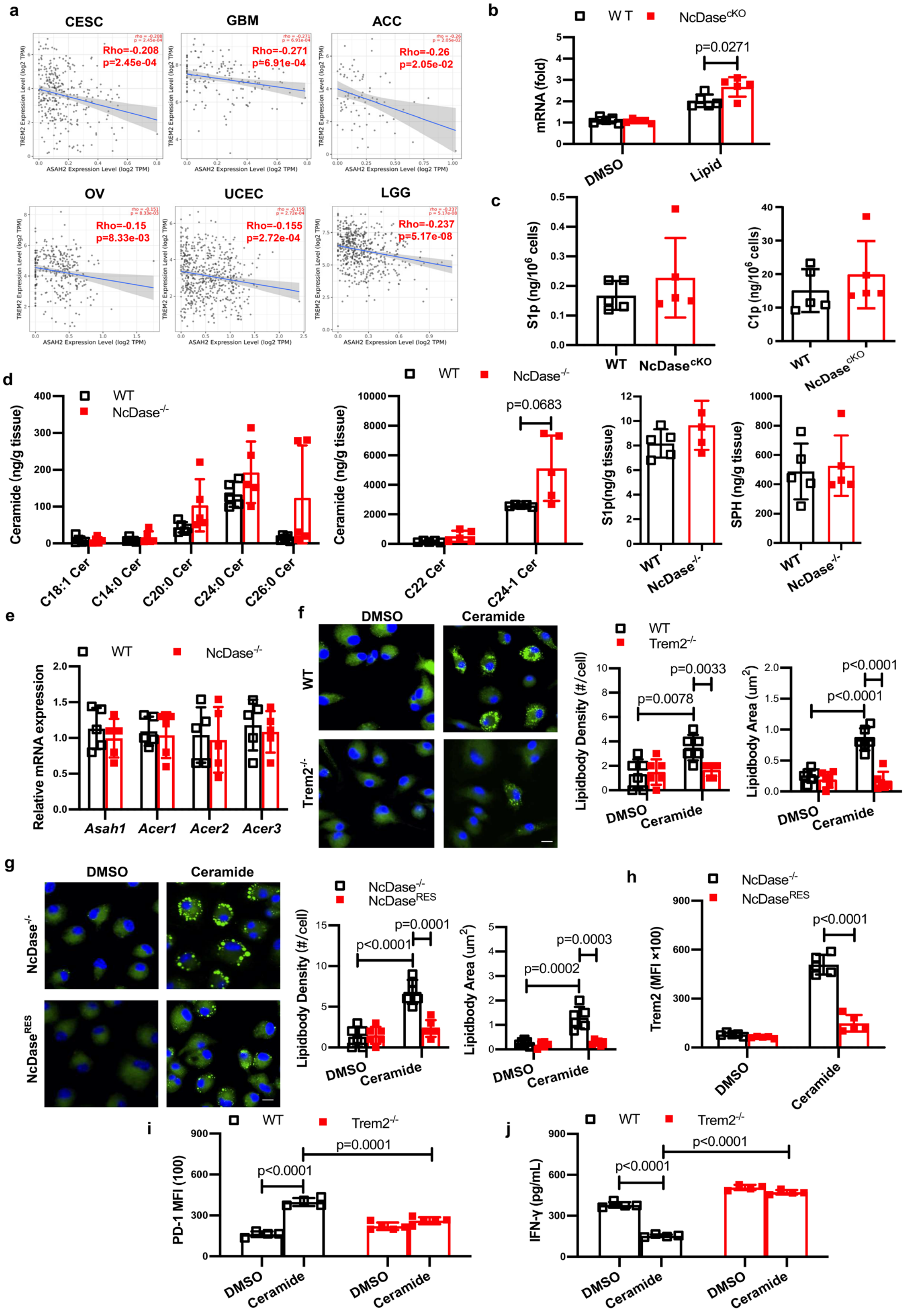

Supplementary Figure 7. Myeloid NcDase contributes to CD8+ T cell exhaustion via TREM2+ TAMs.

(**a**) Correlation between NcDase (*ASAH2*) and *TREM2* expression in human cervical squamous cell carcinoma (CESC, n=306), glioblastoma multiforme (GBM, n=153), adenoid cystic carcinoma (ACC, n=79), ovarian cancer (OV, n=303), uterine corpus endometrial carcinoma (UCEC, n=545), low-grade gliomas (LGGs, n=516).

(**b**) Real-time PCR analysis of the *TREM2* mRNA expression in BMDMs treated for 48 hours with DMSO or tumor lipids. n=5 independent biological samples.

(**c**) Levels of S1p and C1p in CD11b$^+$ F4/80$^+$ TAMs isolated from WT and NcDase^{cKO} mice bearing EO771 tumors. n=5 independent biological samples.

(**d**) Levels of acyl-chain ceramides, S1p and sphingosine (SPH) in tumor tissues from MMTV-PyMT WT and NcDase$^{-/-}$ mice. n=5 independent biological samples.

(**e**) Real-time PCR analysis of the *Asah1*, *Acer1*, *Acer2* and *Acer3* mRNA expression in CD11b$^+$ F4/80$^+$ TAMs isolated from WT PyMT and NcDase$^{-/-}$ PyMT mice. n=5 independent biological experiments.

(**f**) Confocal images and quantification of lipid droplets using BODIPY493/503 staining in DMSO or ceramide-derived BMDMs from WT and TREM2$^{-/-}$ mice. n=6 independent biological samples.

(**g-h**) NcDase$^{-/-}$ BMDMs without or with NcDase overexpression (NcDase^{RES}) were treated with DMSO or ceramide. Quantification of lipid droplets by BODIPY493/503 staining and Confocal analysis (g) and real-time PCR analysis of the *TREM2* mRNA expression (h). (g-h) The total number of LDs was counted per single cell. LD size defined by their cross-sectional area was calculated. n=6 (g) and 5 (h) independent biological experiments.

(**i**) The levels of PD-1 on CD8$^+$ T cells that were co-cultured with BMDMs. BMDMs were derived from WT or TREM2$^{-/-}$ mice and pretreated with DMSO or ceramide. n=4 (WT) and 5 (TREM2$^{-/-}$) independent biological experiments.

(**j**) Quantification of IFN-γ in the supernatant from CD8 T cells/macrophages co-culture experiments. CD8$^+$ T cells were co-cultured with BMDMs derived from WT or TREM2$^{-/-}$ mice and pretreated with DMSO or ceramide. n=4 independent biological experiments.

Statistical comparisons were performed using Pearson's correlation coefficient (PCC) (a), two-way ANOVA with Sidak's multiple comparisons test (b, f, g, h, i, j), two-tailed unpaired t-test (c, d, e). Error bars indicate mean ± SD. Source data are provided as a Source Data file.

Supplementary Table 1. Primers used for Real-time PCR

Gene name	Forward primer	Reverse primer
Gapdh	AGGTCATCCCAGAGCTGAACG	ACCCTGTTGCTGTAGCCGTAT
β-Actin	ACGGCCAGGTCATCACTATTC	AGGAAGGCTGGAAAAGAGCC
Tnf-α	TCTATGGCCCAGACCCTCAC	GACGGCAGAGAGGAGGTTGA
IL-1β	GCAACTGTTCCTGAACTCAACT	ATCTTTTGGGGTCCGTCAACT
Dgat2	GCGCTACTTCCGAGACTACTT	GGGCCTTATGCCAGGAAACT
Dgat1	TCCGTCCAGGGTGGTAGTG	TGAACAAAGAATCTTGCAGACGA
Acsl4	CTCACCATTATATTGCTGCCTGT	TCTCTTTGCCATAGCGTTTTTCT
Acsl5	TCCTGACGTTTGGAACGGC	CTCCCTCAATCCCCACAGAC
Gpat4	AGCTTGATTGTCAACCTCCTG	CCGTTGGTGTAGGGCTTGT
Mgat2	CGGAAGGTGCTAATCCTGACG	CCGATTCGTTTGAGACCCTG
Lipa	TGTTCGTTTTCACCATTGGGA	CGCATGATTATCTCGGTCACA
Lipe	CCAGCCTGAGGGCTTACTG	CTCCATTGACTGTGACATCTCG
Lipc	ATGGGAAATCCCCTCCAAATCT	GTGCTGAGGTCTGAGACGA
Lipg	ATGCGAAACACGGTTTTCCTG	GTAGCTGGTACTCCAGTGGG
Gpat3	GGCCTTCGGATTATCCCTGG	CTTGGGGGCTCCTTTCTGAA
Hilpda	TGCTGGGCATCATGTTGACC	TGACCCCTCGTGATCCAGG
Fizz1	CCTGCTGGGATGACTGCTA	TGGGTTCTCCACCTCTTCAT
Atgl	CCAACACCAGCATCCAGTTCA	CTCCAGCGGCAGAGTATAGGG
Ym1	GCCACTGAGGTCTGGGATGC	TCCTTGAGCCACTGAGCCTTC
Trem2	CAGCACCTCCAGGAATCAAGA	AGGATCTGAAGTTGGTGCCC
Arg-1	CTCCAAGCCAAAGTCCTTAGAG	AGGAGCTGTCATTAGGGACATC

Supplementary Figure 8

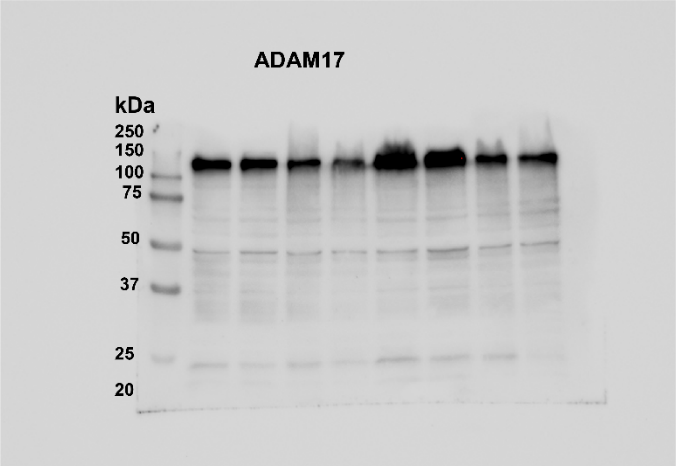

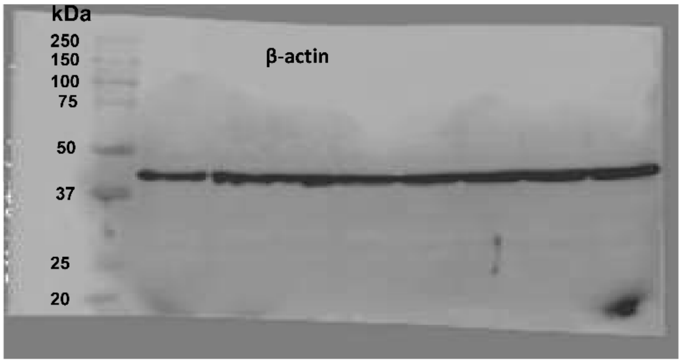

Supplementary Figure 8. Un-cropped western blots represented in Figure 7h.
