# Supplementary material for: Neutral ceramidase regulates breast cancer progression by metabolic programming of TREM2-associated macrophages
Source: Nat Commun. 2024 Feb 1;15:966. doi: 10.1038/s41467-024-45084-7 (PMC10834982; doi:10.1038/s41467-024-45084-7)
Supplement: Supplementary file 3 — Reporting Summary [file 41467_2024_45084_MOESM3_ESM.pdf]

nature portfolio

Corresponding author(s): Zhongbin Deng, Ph.D

Last updated by author(s): Dec 12, 2023

Reporting Summary

Nature Portfolio wishes to improve the reproducibility of the work that we publish. This form provides structure for consistency and transparency in reporting. For further information on Nature Portfolio policies, see our Editorial Policies and the Editorial Policy Checklist.

Statistics

For all statistical analyses, confirm that the following items are present in the figure legend, table legend, main text, or Methods section.

n/a	Confirmed	
☐	☒	The exact sample size (*n*) for each experimental group/condition, given as a discrete number and unit of measurement
☒	☐	A statement on whether measurements were taken from distinct samples or whether the same sample was measured repeatedly
☐	☒	The statistical test(s) used AND whether they are one- or two-sided<br>*Only common tests should be described solely by name; describe more complex techniques in the Methods section.*
☒	☐	A description of all covariates tested
☐	☒	A description of any assumptions or corrections, such as tests of normality and adjustment for multiple comparisons
☐	☒	A full description of the statistical parameters including central tendency (e.g. means) or other basic estimates (e.g. regression coefficient) AND variation (e.g. standard deviation) or associated estimates of uncertainty (e.g. confidence intervals)
☐	☒	For null hypothesis testing, the test statistic (e.g. *F*, *t*, *r*) with confidence intervals, effect sizes, degrees of freedom and *P* value noted<br>*Give P values as exact values whenever suitable.*
☒	☐	For Bayesian analysis, information on the choice of priors and Markov chain Monte Carlo settings
☒	☐	For hierarchical and complex designs, identification of the appropriate level for tests and full reporting of outcomes
☐	☒	Estimates of effect sizes (e.g. Cohen's *d*, Pearson's *r*), indicating how they were calculated

Our web collection on statistics for biologists contains articles on many of the points above.

Software and code

Policy information about availability of computer code

Data collection	FACS DIVA version 6 was used to collect all flow data,IMC images were acquired using Hyperion Imaging Mass Cytometer. CyToF were performed with a Helios Mass Cytometer .
Data analysis	Flowjo v10.7.1 to analyze flow data and data were plotted using tSNE or UMAP analysis for CyTOF analysis. Prism 8 for statistical analysis, Associations between NcDase (ASAH2) mRNA expression and infiltration of different cell types from the TME were analyzed by using xCell2, QUANTISEQ, CIBERSORT and TIMER2.0.

For manuscripts utilizing custom algorithms or software that are central to the research but not yet described in published literature, software must be made available to editors and reviewers. We strongly encourage code deposition in a community repository (e.g. GitHub). See the Nature Portfolio guidelines for submitting code & software for further information.

Data

Policy information about availability of data

All manuscripts must include a data availability statement. This statement should provide the following information, where applicable:

- Accession codes, unique identifiers, or web links for publicly available datasets
- A description of any restrictions on data availability
- For clinical datasets or third party data, please ensure that the statement adheres to our policy

The data generated in this study are available in the data source files. RNAseq data has been uploaded to NIH SRA database (https://www.ncbi.nlm.nih.gov/sra) under accession number PRJNA936597.

Human research participants

Policy information about studies involving human research participants and Sex and Gender in Research.

Reporting on sex and gender	No human research participants
Population characteristics	No human research participants
Recruitment	No human research participants
Ethics oversight	No human research participants

Note that full information on the approval of the study protocol must also be provided in the manuscript.

Field-specific reporting

Please select the one below that is the best fit for your research. If you are not sure, read the appropriate sections before making your selection.

[x] Life sciences [ ] Behavioural & social sciences [ ] Ecological, evolutionary & environmental sciences

For a reference copy of the document with all sections, see nature.com/documents/nr-reporting-summary-flat.pdf

Life sciences study design

All studies must disclose on these points even when the disclosure is negative.

Sample size	Sample size was determined by pilot experiments and power analysis.
Data exclusions	No data exclusions
Replication	All results were repeated at least three independent times unless otherwise specified. Similar data were obtained in independent experiments.
Randomization	Biological samples (cultured cells and tissue extracts) and animals (mice) were allocated randomly into the different groups.
Blinding	Investigators were blinded to group allocation during data collection and analysis. But blinding is not possible in the experiments of treatment since the treatment effect was so obvious.

Reporting for specific materials, systems and methods

We require information from authors about some types of materials, experimental systems and methods used in many studies. Here, indicate whether each material, system or method listed is relevant to your study. If you are not sure if a list item applies to your research, read the appropriate section before selecting a response.

Materials & experimental systems

n/a	Involved in the study	
	x	Antibodies
	x	Eukaryotic cell lines
x		Palaeontology and archaeology
	x	Animals and other organisms
x		Clinical data
x		Dual use research of concern

Methods

n/a	Involved in the study	
x		ChIP-seq
	x	Flow cytometry
x		MRI-based neuroimaging

Antibodies

Antibodies used

Ly6G (141 Pr) Fluidigm Cat# 3141008B; RRID: AB_2814678
CD11c (142 Nd) Fluidigm Cat# 3142003B; RRID: AB_2814737
CD8a (168Er) Fluidigm Cat# 3168003B; RRID: AB_2811241
CD11b (172Yb) Fluidigm Cat# 3172012B; RRID: AB_2661809
CD19 (149 Sm) Fluidigm Cat# 3149002B; RRID: AB_2814679
CD25 (151 Eu) Fluidigm Cat# 3151007B; RRID: AB_2827880
F4/80 (146 Nd) Fluidigm Cat# 3146008B; RRID: AB_2895117
CD36 (147 Sm) Fluidigm Cat# 3147018B;RRID:N/A
CD3e (152 Sm) Fluidigm Cat# 3152004B; RRID: AB_2687836
CD274 (153Eu) Fluidigm Cat# 3153016B; RRID: AB_2687837
CD62L (160 Gd) Fluidigm Cat# 3160008B; RRID: AB_2687840
CD73 (154 Sm) Fluidigm Cat# 3154019B; RRID: AB_2813854
Ly6c (150Nd) Fluidigm Cat# 3150010B; RRID: AB_2895118
CX3CR1 (164 Dy) Fluidigm Cat# 3164023B; RRID: AB_2832247
CD103 (163 Dy) This study N/A
CD206 (169 Tm) Fluidigm Cat# 3169021B; RRID: AB_2832249
NK1.1 (170 Er) Fluidigm Cat# 3170002B; RRID: AB_2885023
CD44 (162Dy) Fluidigm Cat# 3162030B; RRID: AB_2814898
CD4 (145Nd) Fluidigm Cat# 3145002B; RRID: AB_2687832
MHCII (209Bi) Fluidigm Cat# 3209006B; RRID: AB_2885025
CD45 (89Y) Fluidigm Cat# 3089005B; RRID: AB_2651152
B220 (176 Yb) Fluidigm Cat# 3176002B; RRID: AB_2895123
All flow cytometer antibodies were obtained from Thermo Fisher Scientific except specified.
PerCP-Cy5.5 labeled anti-IL-17A (TC11-18H10.1, Biolegend), 506943
PE- labeled anti-IL-17A (TC11-18H10.1, Biolegend), 506903
FITC- labeled anti-IL-17A (TC11-18H10.1), A15377
APC-labeled anti-IL-17A (TC11-18H10.1, Biolegend), cat# 506915
PE- labeled anti-IL-4 (11B11), Cat# 12-7101-41;
APC-labeled anti-IL-4 (11B11) Cat# 17-7041-82;
PE--labelled anti-IL-10 (JES5–16E3) Cat# 12-7101-82
APC-labelled anti-IL-10 (JES5–16E3), Cat# 17-7101-82
APC- labeled anti-IFN-γ (XMG1.2), Cat# 17-7311-82
PE-Cy7-labeled anti-IFN-γ (XMG1.2), Cat# 25-7311-82
PE-labeled anti-Foxp3 (FJK-16s), Cat# 12-5773-82
PE- labeled anti-CD11b (M1/70), Cat# 12-0112-82
FITC- labeled anti-CD11b (M1/70), Cat# 12-0112-82
APC-labeled anti-CD11b (M1/70), Cat# 17-0112-82
PE- labeled anti-CD4 (RM4-5), Cat# 12-0042-82
FITC- labeled anti-CD4 (RM4-5), Cat# 12-0042-82
APC-labeled anti-CD4 (RM4-5), Cat# 17-0042-82
PE-Cy7-labeled anti-CD3 (145-2C11), Cat# 25-0031-82
PE-anti-Gr-1 (RB6-8C5), Cat# 12-5931-82
PE- labeled anti-mouse Ly6G (1A8), Cat# 12-9668-82
FITC-labeled anti-mouse Ly6G (1A8), Cat# 11-9668-82
APC-conjugated CD45.2 (104), Cat# 17-0454-82
PE-conjugated anti-CD45.1 (A20), Cat# 12-0453-82
FITC- labeled anti-mouse Ly6G (1A8), Cat# 11-9668-82
PerCP-Cy5.5 labeled anti-mouse Ly6G (1A8), Cat# 11-9668-82
APC-anti-Lag-3 (C9B7W), Cat# 17-2231-82
PE-anti-CD244.2 (2B4), Cat# 12-2441-82
PE-anti-TOX (TXRX10), Cat# 12-6502-82
PE-anti-CD39 (24DM1), Cat# 25-0391-82
PE-anti-Trem2 (237920, R&D), Cat# FAB17291P

PerCP-Cy5.5, PE-, FITC- or APC-labeled anti-IL-17A (TC11-18H10.1, 1:100), PE- or APC-labeled anti-IL-4 (11B11, eBioscience, Thermo fisher, 1:200), PE- or APC-labelled anti-IL-10 (JES5–16E3, 1:100), APC- or PE-Cy7-labeled anti-IFN- (XMG1.2, 1:200), PE-labeled anti-Foxp3 (FJK-16s, eBioscience, Thermo fisher), PE-, FITC- or APC-labeled anti-CD11b (M1/70, 1:300), PE-, FITC- or APC-labeled anti-CD4 (RM4-5, 1:300), PE-Cy7-labeled anti-CD3 (145-2C11, 1:100), PE-anti-Gr-1 (RB6-8C5, 1:600), PE- or FITC-labeled anti-mouse Ly6G (1A8, 1:200), APC-conjugated CD45.2 (104, 1:400), PE-conjugated anti-CD45.1 (A20, 1:400), FITC-, PerCP-Cy5.5 or Pacific Blue-labelled anti-CD45 (30-F11, 1:400), APC-anti-Lag-3 (C9B7W, 1:100), PE-anti-CD244.2 (2B4, 1:100), PE-anti-TOX (TXRX10, 1:100), APC-anti-CD39 (24DM1, 1:100),

Validation

All antibodies were validated for their use in the application and species according to manufacturer's websites. IHC antibodies were validated as compared to an isotype control. Flow antibodies were validated by titration.

Eukaryotic cell lines

Policy information about cell lines and Sex and Gender in Research

Cell line source(s)	E0771 and Raw264.7 were obtained from ATCC.
Authentication	IDEXX BioResearch company helps check for cell line contamination with cell lines of other species
Mycoplasma contamination	Cells were confirmed mycoplasma negative using ATCC Mycoplasma detection kit
Commonly misidentified lines (See ICLAC register)	None of the cell lines used in the study is listed as Commonly misidentified lines.

Animals and other research organisms

Policy information about studies involving animals; ARRIVE guidelines recommended for reporting animal research, and Sex and Gender in Research

Laboratory animals	MMTV-PyMT mice [B6. FVB/N-Tg(MMTV-PyVT)634Mul/J (Stock No: 022974, Age: 6 week - 1 year) , C57BL/6 mice (6-7 week), Trem2-/- mice and Rag1-deficient mice (Rag1-/-) mice were obtained from Jackson Laboratory. To generate myeloid cell-specific NcDase-deleted mice, NcDasefl/fl mice were first generated using CRISPR/Cas9 technology by Biocytogen and then bred with LysM-cre mice (Jackson Laboratory) to generate control NcDasefl/fl mice and LysM-cre NcDasefl/fl cKO mice (designated as NcDasecKO). NcDase global knockout mice (NcDase-/-) were from Dr. Yusuf A. Hannun (Stony Brook University) and had been backcrossed at least 8 generations to C57BL/61. NcDase-/- mice were crossed with MMTV-PyMT mice or Rag1-/- mice to generate NcDase-/- PyMT mice or Rag1-/-NcDase-/- mice. WT PyMT and NcDase-/- PyMT animals used for breast cancer experiments were female littermates. All mice were housed under SPF condition with free food and water supply with 12 h dark/light cycle at room temperature with controlled humidity (around 55%).
Wild animals	No wild animals was used in this study.
Reporting on sex	Only female mice have been used for this study due to the nature of the tumor studied.
Field-collected samples	No samples were from field.
Ethics oversight	The university of Louisville IACUC committee approved this study.

Note that full information on the approval of the study protocol must also be provided in the manuscript.

Flow Cytometry

Plots

Confirm that:

- [x] The axis labels state the marker and fluorochrome used (e.g. CD4-FITC).
- [x] The axis scales are clearly visible. Include numbers along axes only for bottom left plot of group (a 'group' is an analysis of identical markers).
- [x] All plots are contour plots with outliers or pseudocolor plots.
- [x] A numerical value for number of cells or percentage (with statistics) is provided.

Methodology

Sample preparation	Tumor infiltrating cells were isolated from both subcutaneous and autochthonous tumors at the indicated time points. Briefly, tumor tissues from sacrificed mice were prepared by mechanical disruption followed by digestion for 45 min with collagenase I (Worthington Biochemical, LS004197) and DNase I (1 mg/ml; Roche, 11284932001) at 37°C. Digested tissues were incubated 5 min at 37°C with EDTA (0.5 M) to prevent DC/T cell aggregates and mashed through filters.Viability dye was added to distinguish dead cells, these has been described in depth in the methods section.
Instrument	XF-96 Extracellular Flux Analyzer, FACSAria II, FACS cantoII cytometer (BD Bioscience)
Software	Flowjo v10.7.1 to analyze flow data, prism 8 for statistical analysis, Associations between NcDase (ASAH2) mRNA expression and infiltration of different cell types from the TME were analyzed by using xCell2, CIBERSORT, and TIMER2.0.
Cell population abundance	If sorting was used, purify was always > 90%, these has been described in the methods section.
Gating strategy	We used viability dye to gate out dead cells and then use CD45 to gate all immune cells, then different subsets of cells were gated from CD45+ cells.

☒ Tick this box to confirm that a figure exemplifying the gating strategy is provided in the Supplementary Information.
